# A clinical and biomarker model for predicting post-stroke cognitive impairment: a prospective cohort study

**DOI:** 10.3389/fneur.2026.1963734

**Published:** 2026-10-14

**Authors:** Xinyu Zhang, Xiangwen Hao, Yanyang Li, Jingqi Chen, Xiaoyan Zheng, Anren Zhang, Qiuhong Man

**Affiliations:** 1 School of Health Preservation and Rehabilitation, Chengdu University of Traditional Chinese Medicine, Chengdu, China; 2 Department of Clinical Laboratory, Shanghai Fourth People's Hospital, School of Medicine, Tongji University, Shanghai, China; 3 Department of Rehabilitation Medicine, Shanghai Fourth People's Hospital Affiliated to Tongji University, Shanghai, China

**Keywords:** blood biomarkers, cognitive impairment, ischemic stroke, post-stroke cognitive impairment, risk prediction

## Abstract

**Background and objectives:**

Post-stroke cognitive impairment (PSCI) is highly prevalent and substantially compromises functional recovery and quality of life; however, early identification remains challenging. This study aimed to develop and validate an early prediction model for PSCI by integrating routinely available blood biomarkers with clinical characteristics.

**Methods:**

Patients with acute ischemic stroke were prospectively enrolled at Shanghai Fourth People's Hospital between March and November 2024. Cognitive function was assessed 3 months after stroke using the Telephone Montreal Cognitive Assessment (T-MoCA), a telephone-based cognitive screening instrument, with PSCI defined as a corrected T-MoCA score <19. Univariable logistic regression, Spearman correlation analysis, and variance inflation factor analysis were performed to characterize associations and predictor redundancy. Bootstrap least absolute shrinkage and selection operator (LASSO) logistic regression with 1,000 resampling iterations was used to assess variable-selection stability, followed by multivariable logistic regression and nomogram construction. Model performance was evaluated using receiver operating characteristic (ROC) analysis, calibration analysis, decision curve analysis (DCA), and clinical impact curves (CIC), with 1,000 bootstrap iterations used for internal validation.

**Results:**

A total of 198 patients were included, of whom 138 developed PSCI and 60 were classified as post-stroke cognitively normal (PSCN, non-PSCI group). The final prediction model incorporated age, prior stroke history, diabetes mellitus, small dense low-density lipoprotein (sdLDL), apolipoprotein A-I (ApoA-I), homocysteine, cystatin C, and D-dimer. The model achieved an apparent AUC of 0.798. Following 1,000 bootstrap resampling iterations, the mean AUC was 0.777 (95% CI, 0.733–0.802), and the optimism-corrected AUC was 0.762. Calibration analysis suggested reasonable agreement between predicted and observed risks, while DCA and CIC analyses indicated potential utility for PSCI risk stratification.

**Conclusions:**

This study developed and internally validated a multivariable model combining clinical characteristics and circulating biomarkers to estimate PSCI risk after acute ischemic stroke. The model showed acceptable predictive performance and may support early risk stratification.

## 1 Introduction

Ischemic stroke is caused primarily by an interruption in cerebral blood flow and represents one of the leading causes of mortality and disability worldwide. Post-stroke cognitive impairment (PSCI) is a common and serious complication thatsubstantially affects patient outcomes (1, 2). Studies report that 20% to 80% of stroke survivors develop cognitive impairment within three months, with variation largely due to differences in assessment methods and stroke characteristics (3, 4). PSCI typically manifests as visuospatial dysfunction, executive dysfunction, and memory impairment. These deficits not only impair daily functioning and mental health but also significantly worsen prognosis. It has been reported that patients with PSCI have a 59% higher risk of recurrent stroke and double the mortality rate compared to those without cognitive impairment (5–7).

Despite its clinical importance, PSCI remains difficult to predict. The preclinical period of PSCI can last up to 6–12 months, during which symptoms may develop insidiously, while current diagnostic and predictive approaches remain limited. Cognitive screening commonly relies on the Mini-Mental State Examination (MMSE) and the Montreal Cognitive Assessment (MoCA). Salvadori et al. (8) demonstrated that MoCA performance assessed during the acute phase after stroke was predictive of PSCI at 6–9 months. Zietemann et al. (9) further showed that early MoCA performance was associated with long-term cognitive and functional outcomes after stroke. In contrast, Bour et al. (10) reported limitations in the use of the MMSE for predicting subsequent cognitive performance after stroke. Neuroimaging may provide complementary prognostic information; for example, Zhang et al. found that infarct location, including thalamic and right-hemispheric lesions, was associated with subsequent cognitive decline (11). However, cognitive screening scales and neuroimaging techniques have distinct limitations. Cognitive assessments may be influenced by educational background, language and communication difficulties, and patient-related factors, whereas neuroimaging may be constrained by cost, accessibility, examination time, and, for CT-based techniques, exposure to ionizing radiation. These limitations may restrict their routine use as stand-alone tools for early PSCI risk prediction.

Blood biomarkers offer a promising approach for the early prediction of post-stroke cognitive impairment owing to their ease of sampling, high patient acceptability, and cost-effectiveness. Previous studies revealed that lipid metabolism dysfunction plays a key role in PSCI development (12). Abnormal lipid metabolism contributes to PSCI through multiple mechanisms, including atherogenic lipids [oxidized LDL, lipoprotein(a)] damage blood vessels, neuroinflammation increases, and neural tissue suffers direct injury (13–15). Zhang et al. (16) demonstrated that mangiferin attenuates post-stroke cognitive impairment in a rat model of cerebral ischemia–reperfusion by modulating 13 lipid metabolites. Li et al. (17) found that astrocytic endothelin-1 overexpression impairs hippocampal neurogenesis, learning, and memory in ischemic stroke mice by altering lipid metabolites such as glycerophosphatidylcholine, myelin sheath proteins, and phosphatidic acid. Clinical evidence aligns with these findings, lower HDL levels correlate with reduced phosphorylated α-synuclein and higher PSCI rates (18), and 2-hydroxypropyl-β-cyclodextrin (HPβCD) prevents cognitive decline by reducing lipid accumulation and inflammation (19). These findings establish lipid metabolism as central to PSCI pathophysiology. However, individual lipid markers lack specificity because they are involved in many disease states, limiting their predictive value when used alone.

Related analyses from the same prospective research program have been reported previously. Although some participants may overlap, these studies differed in research objectives, eligibility criteria, and biomarker panels. The present study focuses on combining routinely available lipid-related biomarkers with clinical characteristics for PSCI risk prediction.

Given the need for accessible and accurate PSCI prediction tools, this study aims to integrate blood biomarkers with clinical characteristics to identify potential blood biomarkers for early PSCI screening and to develop a predictive model enabling early detection and accurate risk stratification.

## 2 Materials and methods

### 2.1 Patients

A total of 256 patients with acute ischemic stroke admitted to Shanghai Fourth People's Hospital between March 2024 and November 2024 were consecutively screened for eligibility. Ischemic stroke was confirmed by computed tomography (CT) or magnetic resonance imaging (MRI). After application of the predefined eligibility criteria and exclusion of patients who did not complete the 3-month cognitive assessment or follow-up, 198 patients were included in the final analysis. Participants were subsequently classified into the post-stroke cognitive impairment (PSCI) group (*n* = 138) and the post-stroke cognitively normal (PSCN) group (*n* = 60) according to the 3-month T-MoCA assessment. The patient selection process is summarized in Figure 1. The study population consisted of consecutive patients with acute ischemic stroke treated at our institution, reflecting a real-world clinical cohort. However, as this was a single-center cohort, the observed proportion of PSCI may have been influenced by local referral patterns, patient characteristics, and the requirement for completion of 3-month cognitive assessment. Therefore, the event rate should be interpreted within the context of this specific cohort rather than as a population-based incidence estimate. Although this approach may enhance the representativeness of the enrolled population, the single-center design may limit the extrapolation of findings to other regions and clinical settings.

**Figure 1:**
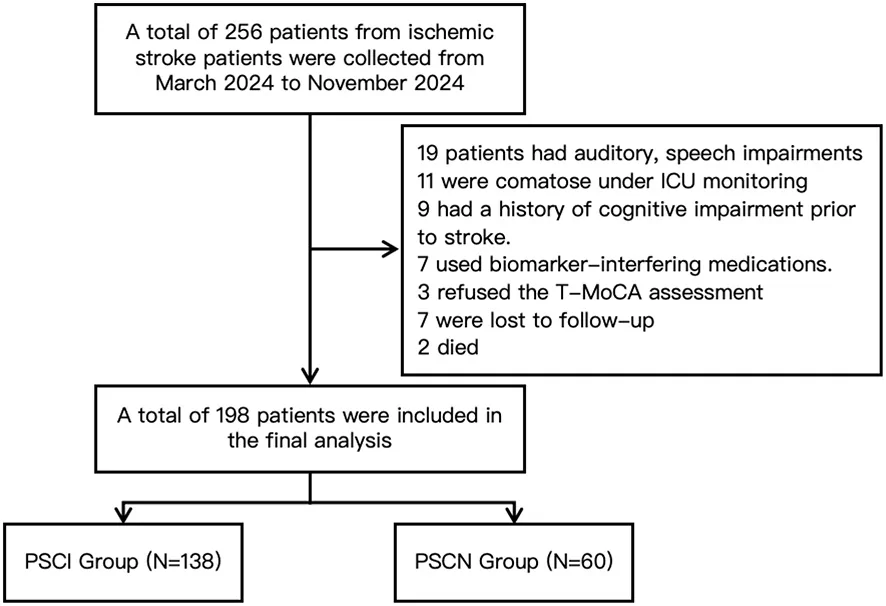
Flowchart for participant selection

The inclusion criteria were as follows: (1) age ≥18 years; (2) ischemic stroke confirmed by CT or MRI; (3) admission within 72 h of symptom onset; (4) availability of the required baseline clinical and laboratory data; (5) a clinically stable condition allowing cognitive follow-up; and (6) written informed consent obtained from the patient or a legal representative.

The exclusion criteria were as follows: (1) brain tumors, traumatic brain injury, or other neurological disorders potentially affecting cognitive function; (2) inability to complete cognitive assessment because of severe aphasia, hearing impairment, impaired consciousness, or severe communication impairment; (3) severe cardiac, hepatic, renal, or respiratory failure; (4) acute infection, malignancy, hematologic disease, autoimmune disease, recent major surgery, or trauma associated with systemic inflammatory or immune activation; (5) use of medications considered likely to substantially interfere with the biomarkers under investigation; (6) inability or refusal to complete the 3-month cognitive assessment; and (7) pregnancy or lactation.

### 2.2 Data collection

Baseline demographic characteristics, medical history, clinical variables, treatment information, and laboratory parameters were collected after admission. Demographic variables included age and sex. Medical history included previous stroke, hypertension, diabetes mellitus, coronary heart disease, dyslipidemia, atrial fibrillation, smoking, alcohol use, and previous surgery. Clinical variables included National Institutes of Health Stroke Scale (NIHSS) and Glasgow Coma Scale (GCS) scores on admission, thrombolysis, thrombectomy, Trial of Org 10172 in Acute Stroke Treatment (TOAST) classification, Oxfordshire Community Stroke Project (OCSP) classification, systolic and diastolic blood pressure, and body mass index (BMI).

Laboratory parameters included small dense low-density lipoprotein (sdLDL), apolipoprotein B (ApoB), apolipoprotein A-I (ApoA-I), apolipoprotein E (ApoE), triglycerides, low-density lipoprotein cholesterol (LDL-C), high-density lipoprotein cholesterol (HDL-C), total cholesterol, homocysteine, folate, vitamin B12, glycated hemoglobin, fasting blood glucose, D-dimer, fibrinogen, cystatin C (CysC), and C-reactive protein (CRP). Fasting venous blood samples were obtained on the morning following admission and before intravenous thrombolytic therapy.

Hypertension, diabetes mellitus, coronary heart disease, and atrial fibrillation were determined according to documented medical history and/or corresponding medication use. Smoking history was defined as smoking at least one cigarette per day for more than 6 months, and alcohol use was defined as alcohol consumption of at least 50 mL per week for more than 6 months. Previous surgery was defined as a documented history of any surgical procedure before the index hospitalization. Dyslipidemia was defined according to a documented pre-existing diagnosis in the medical record before the index admission. BMI was calculated as body weight in kilograms divided by height in meters squared. Stroke severity was evaluated using the NIHSS (20), whereas level of consciousness was assessed using the GCS. Stroke etiology was classified according to the TOAST criteria and clinical stroke syndromes were categorized according to the OCSP classification.

Blood samples were collected before intravenous thrombolysis and processed according to standardized hospital laboratory procedures. Routine biochemical and immunological biomarkers were measured using an Atellica automated chemistry and immunoassay system (Siemens Healthineers, USA), with corresponding commercial reagents, while hematological parameters were measured using a Mindray 7,500 automated hematology analyzer (Mindray, Shenzhen, China). Internal quality-control procedures were performed before testing, and samples were analyzed only when daily quality-control results were within the acceptable range. Quality-control materials were provided by the Shanghai Center for Clinical Laboratory.

### 2.3 Assessment of cognitive function

Cognitive function was assessed 3 months after ischemic stroke using the telephone version of the Montreal Cognitive Assessment (T-MoCA) (21–23), administered by a trained clinician blinded to baseline clinical and laboratory information. The T-MoCA was selected because it provides a standardized assessment of multiple cognitive domains and is particularly suitable for post-stroke follow-up when face-to-face cognitive evaluation may be difficult. In this prospective cohort, T-MoCA was the only cognitive screening instrument used at the 3-month follow-up because of its feasibility, accessibility, and established application in evaluating post-stroke cognitive impairment. No additional cognitive screening instruments, such as the Mini-Mental State Examination (MMSE), were administered. The T-MoCA assesses attention (6 points), language (3 points), abstraction (2 points), delayed recall (5 points), and orientation (6 points), yielding a maximum score of 22 points. Lower scores indicate poorer cognitive performance (24–26). To account for educational attainment, one additional point was added for participants with ≤ 12 years of education and a raw T-MoCA score < 22. PSCI was defined as a corrected T-MoCA score < 19, whereas participants with scores ≥19 were classified as PSCN (23, 24, 27). Participants were permitted to use hearing aids when necessary during the telephone assessment. Although T-MoCA enables standardized telephone-based cognitive screening, it does not include certain visually mediated visuospatial and executive tasks available in face-to-face cognitive assessments. Therefore, T-MoCA was used as a feasible screening measure for identifying cognitive impairment after stroke rather than as a substitute for comprehensive neuropsychological evaluation. Sex was recorded as a baseline demographic characteristic and was included in the clinical assessment. However, handedness was not systematically collected in this cohort and therefore could not be evaluated as a potential influencing factor of cognitive test performance.

### 2.4 Statistical analysis

Statistical analyses and graphical visualization were performed using R software (version 4.5.2). There were no missing data for the variables included in the final analysis; therefore, no imputation was performed. Continuous variables were summarized as mean ± standard deviation, and categorical variables were presented as frequencies and percentages. Between-group differences between the PSCI and PSCN groups were assessed using the Mann–Whitney U test for continuous variables and appropriate categorical tests for categorical variables. A two-sided *P* value < 0.05 was considered statistically significant.

Univariable logistic regression analyses were first performed to examine associations between baseline clinical and laboratory variables and PSCI, with odds ratios (ORs) and 95% confidence intervals (CIs) reported. Spearman rank correlation analysis was used to assess correlations among continuous candidate predictors and exploratory relationships between lipid-related biomarkers and T-MoCA scores. Multicollinearity among candidate predictors was further evaluated using variance inflation factors (VIFs), with a VIF threshold of 5 used to identify potentially important collinearity. Correlation and VIF analyses were used to assess predictor redundancy rather than to select variables solely according to statistical significance.

To improve the stability of variable selection and reduce the influence of sampling variation, bootstrap least absolute shrinkage and selection operator (LASSO) logistic regression was performed using 1,000 bootstrap resamples. The frequency with which each candidate predictor was selected across bootstrap samples was calculated as an indicator of selection stability. Variables demonstrating higher selection stability were considered for subsequent multivariable logistic regression modeling. The final model incorporated age, prior stroke history, diabetes mellitus, sdLDL, ApoA-I, homocysteine, CysC, and D-dimer.

A multivariable logistic regression model was then fitted using the selected predictors, and a nomogram was constructed from the fitted model to provide individualized estimates of PSCI probability. Model discrimination was assessed using receiver operating characteristic (ROC) curves and the area under the ROC curve (AUC). The apparent performance of the combined model was compared descriptively with that of individual predictors.

Internal validation was performed using 1,000 bootstrap resampling iterations. Bootstrap estimates of discrimination were obtained, and optimism was estimated to derive an optimism-corrected AUC. Model calibration was assessed using calibration curves comparing predicted and observed probabilities. Decision curve analysis (DCA) was used to explore net benefit across a range of threshold probabilities, and a clinical impact curve (CIC) was generated to illustrate the relationship between the number of patients classified as high risk and the number of observed PSCI events.

## 3 Results

### 3.1 Baseline characteristics

A total of 198 patients with acute ischemic stroke were included in the analysis. Patients were classified according to the presence of post-stroke cognitive impairment into the PSCI group (*n* = 138) and the non-PSCI (PSCN) group (*n* = 60). Baseline clinical characteristics and laboratory parameters of the two groups are summarized in Table 1. The mean age was 66.52 ± 9.54 years in the PSCI group and 68.93 ± 9.33 years in the PSCN group. In the PSCI group, 44 patients (31.9%) were female and 94 (68.1%) were male, whereas in the PSCN group, 23 patients (38.3%) were female and 37 (61.7%) were male. There were no significant differences between the two groups in sex, age, admission NIHSS and GCS scores, rates of thrombolysis or thrombectomy, TOAST or OCSP classification, systolic and diastolic blood pressure, BMI, most comorbidities, or medication use (all *P* > 0.05). A history of prior stroke was significantly less frequent in the PSCI group than in the PSCN group (*P* < 0.05). Among the laboratory parameters, small dense LDL, apolipoprotein B, triglycerides, and LDL-C were significantly higher in the PSCI group (*P* < 0.05). In contrast, homocysteine levels were significantly lower in the PSCI group than in the PSCN group (*P* < 0.05). No significant between-group differences were observed for apolipoprotein A1, apolipoprotein E, HDL-C, total cholesterol, folate, vitamin B12, glycated hemoglobin, fasting glucose, D-dimer, fibrinogen, cystatin C, or C-reactive protein (*P* > 0.05).

**Table 1:** Baseline characteristics of patients in the PSCI and PSCN groups.

Characteristic	Overall	PSCI	PSCN	*P* value
*N*	198	138	60	
Sex, *n* (%)				0.473
Female	67 (34.10)	44 (31.90)	23 (38.30)	
Male	131 (66.20)	94 (68.10)	37 (61.70)	
Age, years	68.20 (9.44)	66.52 (9.54)	68.93 (9.33)	0.103
Admission NIHSS score	2.47 (3.64)	2.27 (3.49)	2.56 (3.72)	0.598
Admission GCS score	14.79 (1.26)	14.93 (0.52)	14.72 (1.47)	0.142
Thrombolysis, *n* (%)				0.624
No	164 (82.7)	116 (84.1)	48 (80.0)	
Yes	34 (17.20)	22 (15.90)	12 (20.00)	
Thrombectomy, *n* (%)				0.708
No	188 (94.90)	130 (94.20)	58 (96.70)	
Yes	10 (5.10)	8 (5.80)	2 (3.30)	
TOAST classification, *n* (%)				0.053
Large-artery atherosclerosis	143 (72.20)	97 (70.30)	46 (76.70)	
Small-vessel occlusion	37 (18.70)	24 (17.40)	13 (21.70)	
Cardioembolism	18 (9.10)	17 (12.30)	1 (1.70)	
OCSP classification, *n* (%)				0.713
Total anterior circulation	50 (25.3%)	37 (26.80)	13 (21.70)	
Partial anterior circulation	83 (41.90%)	57 (41.30)	26 (43.30)	
Posterior circulation	59 (29.80%)	39 (28.30)	20 (33.30)	
Lacunar	6 (3.00%)	5 (3.60)	1 (1.7)	
Systolic BP, mmHg	144.52 (18.90)	145.38 (18.94)	142.52 (18.81)	0.328
Diastolic BP, mmHg	82.34 (10.52)	83.03 (10.36)	80.77 (10.80)	0.165
BMI, kg/m^2^	24.71 (3.72)	24.84 (3.66)	24.39 (3.85)	0.435
Medical history, *n* (%)				
Stroke	24 (12.10)	1 (1.70)	23 (16.70)	0.006
Hypertension	119 (60.10)	40 (66.70)	79 (57.20)	0.277
Diabetes	60 (30.30)	24 (40.00)	36 (26.10)	0.074
Coronary heart disease	17 (8.60)	4 (6.70)	13 (9.40)	0.719
Dyslipidemia	7 (3.50)	1 (1.70)	6 (4.30)	0.603
Atrial fibrillation	7 (3.50)	2 (3.30)	5 (3.60)	1.000
Smoking	25 (12.60)	10 (16.70)	15 (10.90)	0.370
Alcohol use	18 (9.10)	7 (11.70)	11 (8.00)	0.574
Surgery	39 (19.70)	10 (16.70)	29 (21.00)	0.608
Medication use, *n* (%)				
Antiplatelet agents	34 (17.20)	9 (15.00)	25 (18.10)	0.742
Anticoagulants	9 (4.50)	1 (1.70)	8 (5.80)	0.362
Antihypertensive drugs	103 (52.00)	33 (55.00)	70 (50.70)	0.690
Hypoglycemic drugs	52 (26.30)	18 (30.00)	34 (24.60)	0.540
Laboratory parameters				
Small dense LDL, mmol/L	0.99 (0.38)	1.15 (0.39)	0.93 (0.36)	< 0.001
Apolipoprotein B, g/L	0.83 (0.23)	0.91 (0.21)	0.80 (0.22)	0.002
Apolipoprotein A1, g/L	1.19 (0.18)	1.17 (0.15)	1.21 (0.19)	0.124
Apolipoprotein E, mg/dL	4.38 (1.59)	4.49 (1.85)	4.34 (1.47)	0.583
Triglycerides, mmol/L	1.67 (0.99)	1.93 (1.35)	1.56 (0.76)	0.049
LDL-C, mmol/L	2.71 (0.88)	3.01 (0.93)	2.58 (0.83)	0.003
HDL-C, mmol/L	1.08 (0.26)	1.06 (0.23)	1.08 (0.27)	0.501
Total cholesterol, mmol/L	4.44 (1.09)	4.69 (1.22)	4.33 (1.01)	0.053
Homocysteine, μmol/L	17.28 (15.36)	14.48 (5.72)	18.49 (17.89)	0.019
Folate, ng/mL	22.82 (12.45)	24.02 (12.72)	22.30 (12.35)	0.378
Vitamin B12, pmol/L	332.99 (205.87)	297.82 (161.44)	348.28 (221.23)	0.074
Glycated hemoglobin, %	6.85 (1.61)	7.06 (1.75)	6.77 (1.55)	0.274
Fasting glucose, mmol/L	6.04 (2.59)	6.39 (2.61)	5.89 (2.58)	0.217
D-Dimer, mg/L	0.77 (1.07)	0.61 (0.59)	0.85 (1.22)	0.068
Fibrinogen, g/L	3.58 (0.83)	3.75 (0.79)	3.51 (0.84)	0.063
Cystatin C, mg/L	0.98 (0.33)	1.04 (0.28)	0.96 (0.35)	0.104
C-reactive protein,mg/L	8.82 (39.15)	3.71 (6.04)	11.05 (46.60)	0.071

### 3.2 Analysis of clinical features and laboratory variables

Univariable logistic regression analysis was performed to examine the associations between baseline clinical and laboratory variables and PSCI. Seven variables were significantly associated with PSCI. Higher levels of small dense low-density lipoprotein (sdLDL) were associated with higher odds of PSCI (OR = 4.629, *P* < 0.001). Similar positive associations were observed for LDL-C (OR = 1.749, *P* < 0.05), apolipoprotein B (OR = 8.674, *P* = 0.003), triglycerides (OR = 1.434, *P* < 0.05), and total cholesterol (OR = 1.344, *P* < 0.05). In contrast, a history of prior stroke was associated with lower odds of PSCI in the univariable model (OR = 0.085 P < 0.05). TOAST classification was also significantly associated with PSCI, with the cardioembolic subtype showing lower odds relative to the reference category (OR = 0.124 *P* = 0.046). The detailed results are presented in Table 2.

**Table 2:** Univariable logistic regression analysis of factors associated with post-stroke cognitive impairment.

Characteristic	No. of PSCI events/total	OR	OR (95% CI)	*P* value
Small dense LDL	138/198	4.629	4.629 (2.046–10.976)	< 0.001
LDL-C	138/198	1.749	1.749 (1.231–2.533)	0.002
Apolipoprotein B	138/198	8.674	8.674 (2.173–37.876)	0.003
Stroke	138/198	0.085	0.085 (0.005–0.418)	0.017
Triglycerides	138/198	1.434	1.434 (1.065–1.968)	0.020
Total cholesterol	138/198	1.344	1.344 (1.018–1.792)	0.039
TOAST Classification	138/198	0.124	0.124 (0.007–0.633)	0.046

This table presents only variables reaching statistical significance (*P* < 0.05) in univariable logistic regression analyses. Results for all variables examined are shown in Supplementary Table S1.

Exploratory analyses illustrated the relationships of these variables with T-MoCA scores (Figure 2). Spearman correlation analysis among continuous predictors demonstrated substantial correlations between apolipoprotein B and LDL-C (ρ = 0.87), apolipoprotein A1 and HDL-C (ρ = 0.75), and apolipoprotein B and sdLDL (ρ = 0.69). Consistent with these findings, VIF analysis identified collinearity among several lipid-related predictors. Correlation and VIF analyses were therefore used to assess predictor redundancy before model development (Figure 3) and were not used to determine predictor inclusion based on statistical significance.

**Figure 2:**
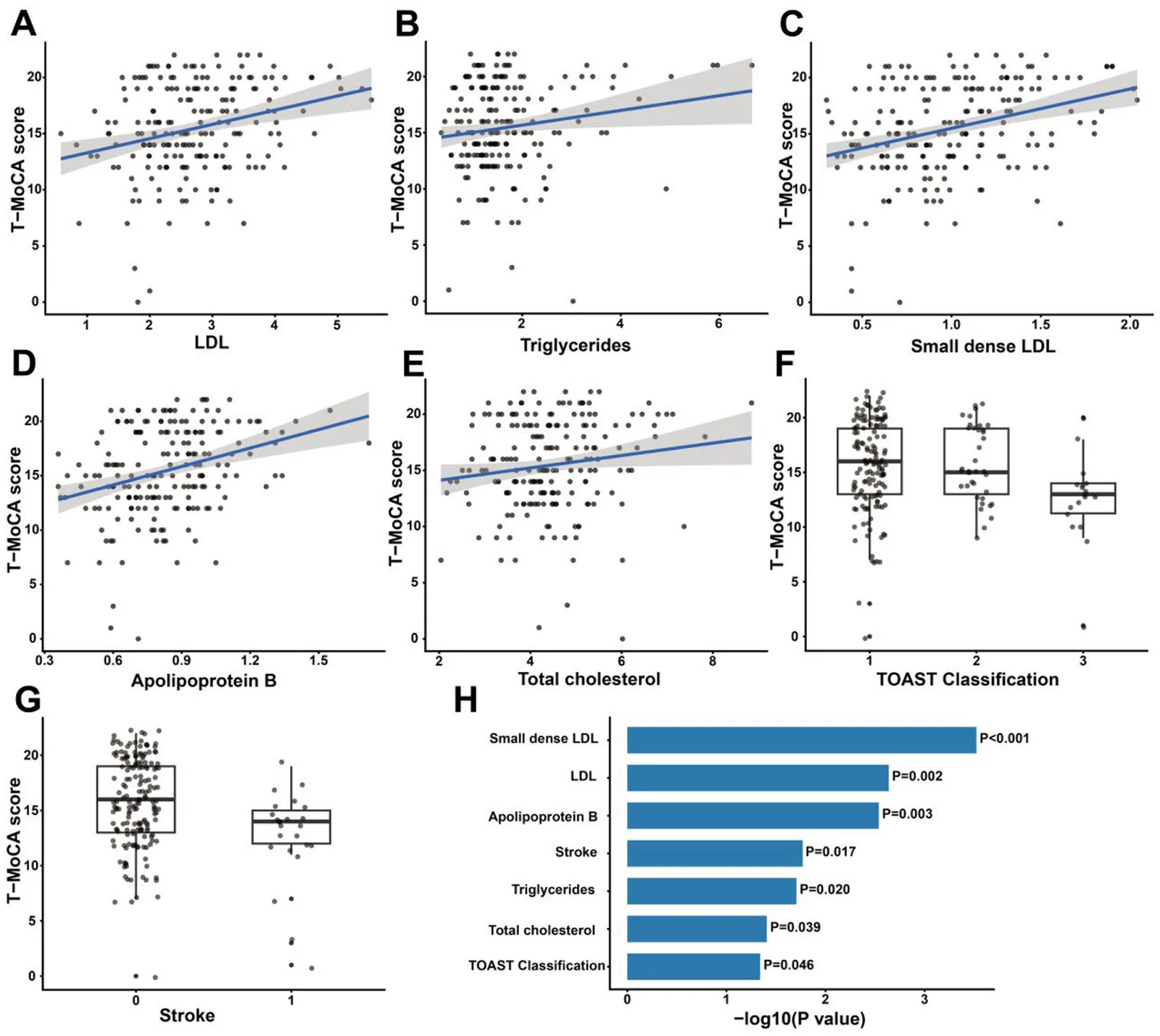
Univariable analysis of factors associated with PSCI and their relationships with T-MoCA scores. **(A–E)** Scatter plots showing the relationships between significant continuous variables and T-MoCA scores. The x-axis represents the measured value or serum concentration of each variable, including LDL-C **(A)**, triglycerides **(B)**, small dense LDL **(C)**, apolipoprotein B **(D)**, and total cholesterol **(E)**, whereas the y-axis represents the T-MoCA score. Solid lines indicate fitted linear regression lines and shaded areas indicate 95% confidence intervals. **(F)** Distribution of T-MoCA scores across TOAST subtypes (1, large-artery atherosclerosis; 2, small-vessel occlusion; 3, cardioembolism). **(G)** Distribution of T-MoCA scores according to stroke history (0, no prior stroke; 1, prior stroke). **(H)** Significant variables from the univariable logistic regression analysis (*P* < 0.05), displayed as –log10(P value); longer bars indicate smaller P values. Abbreviations: T-MoCA, Telephone Montreal Cognitive Assessment; LDL-C, low-density lipoprotein cholesterol; sdLDL, small dense low-density lipoprotein; ApoB, apolipoprotein B; TOAST, Trial of Org 10172 in Acute Stroke Treatment; PSCI, post-stroke cognitive impairment.

**Figure 3:**
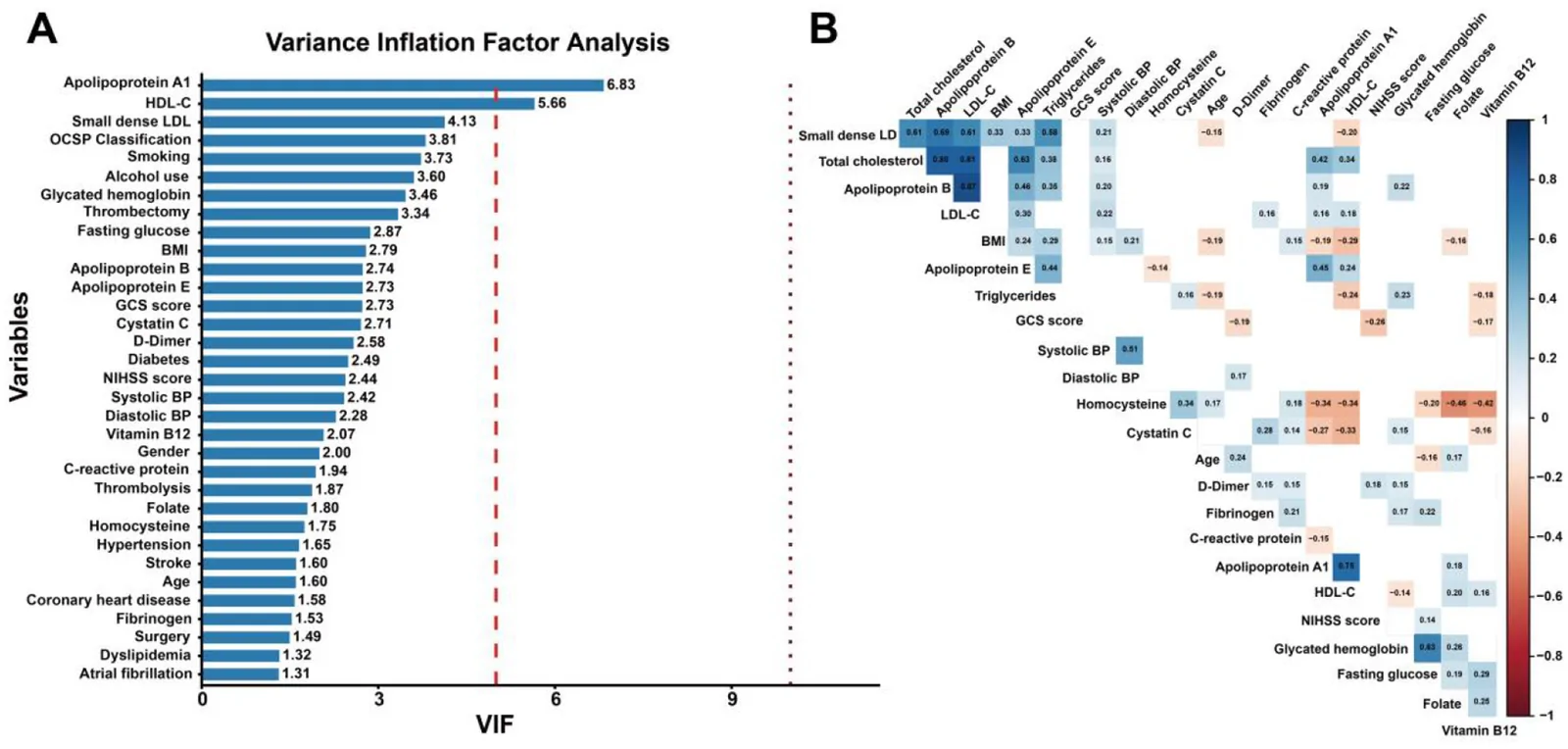
Assessment of multicollinearity and correlations among candidate predictors. **(A)** Variance inflation factor (VIF) analysis of candidate predictors. The x-axis represents the VIF values, and the y-axis represents the corresponding variables. The red dashed line indicates the predefined threshold of VIF = 5. Variables with higher VIF values indicate greater potential collinearity among predictors. **(B)** Spearman correlation matrix showing the correlations among continuous candidate predictors. The values in each cell represent Spearman correlation coefficients (ρ). Blue and red colors indicate positive and negative correlations, respectively, with color intensity representing the strength of correlation. VIF, variance inflation factor.

### 3.3 Predictor selection via bootstrap LASSO logistic regression and multivariable model development

To improve the stability of predictor selection and reduce the potential influence of sampling variation, bootstrap LASSO logistic regression was performed with 1,000 resampling iterations. The selection frequency of each candidate predictor was calculated across bootstrap samples (Figure 4A). Variables demonstrating higher selection stability were retained for subsequent multivariable logistic regression modeling.

**Figure 4:**
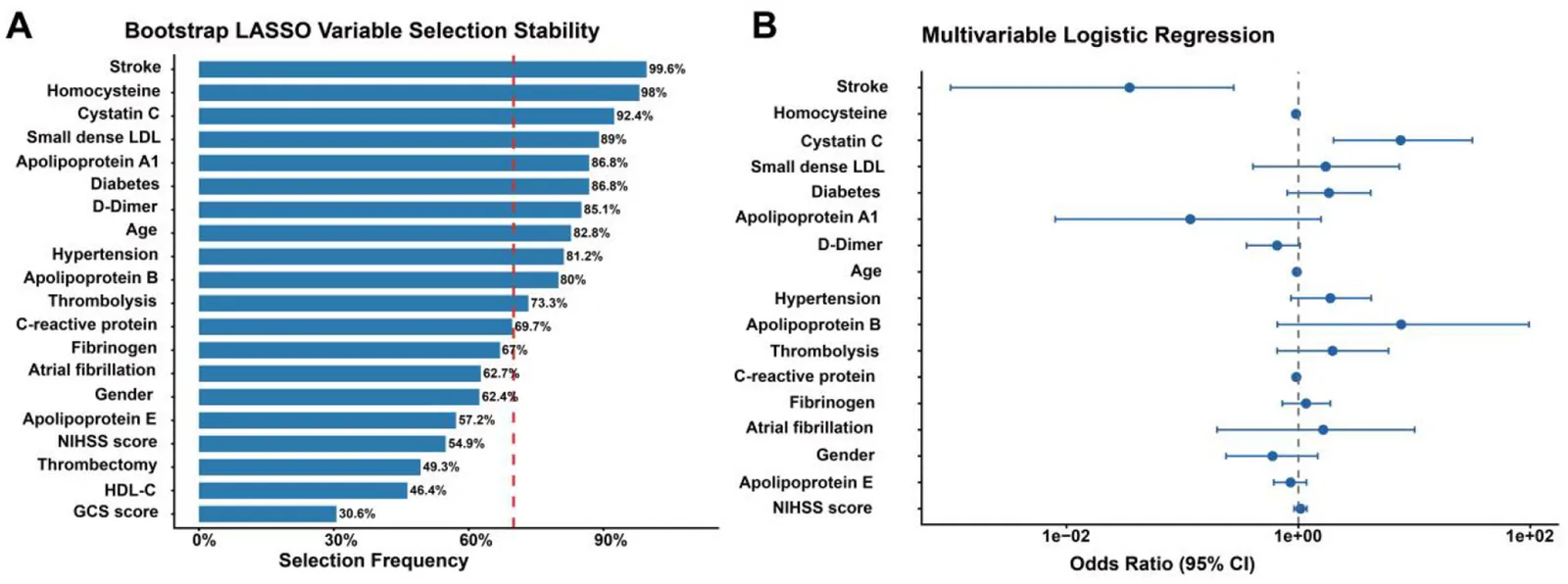
Bootstrap LASSO variable selection stability and multivariable logistic regression model development for predicting PSCI. **(A)** Variable selection stability assessed using bootstrap LASSO logistic regression with 1,000 resampling iterations. The x-axis represents the selection frequency of each candidate predictor, and the y-axis represents the corresponding variables. The red dashed line indicates the predefined selection frequency threshold. Variables with higher selection frequencies demonstrated greater stability across bootstrap samples. **(B)** Forest plot showing the multivariable logistic regression model incorporating the candidate predictors. Odds ratios (ORs) and 95% confidence intervals (CIs) are presented for each variable, with the vertical reference line indicating OR = 1.

The final multivariable logistic regression model included stroke history, homocysteine, cystatin C, sdLDL, diabetes mellitus, apolipoprotein A1, D-dimer, and age (Figure 4B). Among these predictors, cystatin C and sdLDL showed positive associations with PSCI, whereas stroke history and homocysteine showed inverse associations. The estimated odds ratios and 95% confidence intervals for all predictors are presented in Figure 4B.

Although prior stroke history showed an inverse association with PSCI in both univariable and multivariable analyses, this finding should be interpreted cautiously given the observational design and characteristics of the enrolled population.

### 3.4 Development and performance evaluation of the prediction model

Based on the predictors identified through bootstrap LASSO logistic regression and multivariable logistic regression analysis, a prediction model for PSCI was developed incorporating age, stroke history, diabetes mellitus, small dense LDL (sdLDL), apolipoprotein A1, homocysteine, cystatin C, and D-dimer. A nomogram was constructed to provide an individualized estimation of PSCI risk by integrating these selected predictors (Figure 5A).

**Figure 5:**
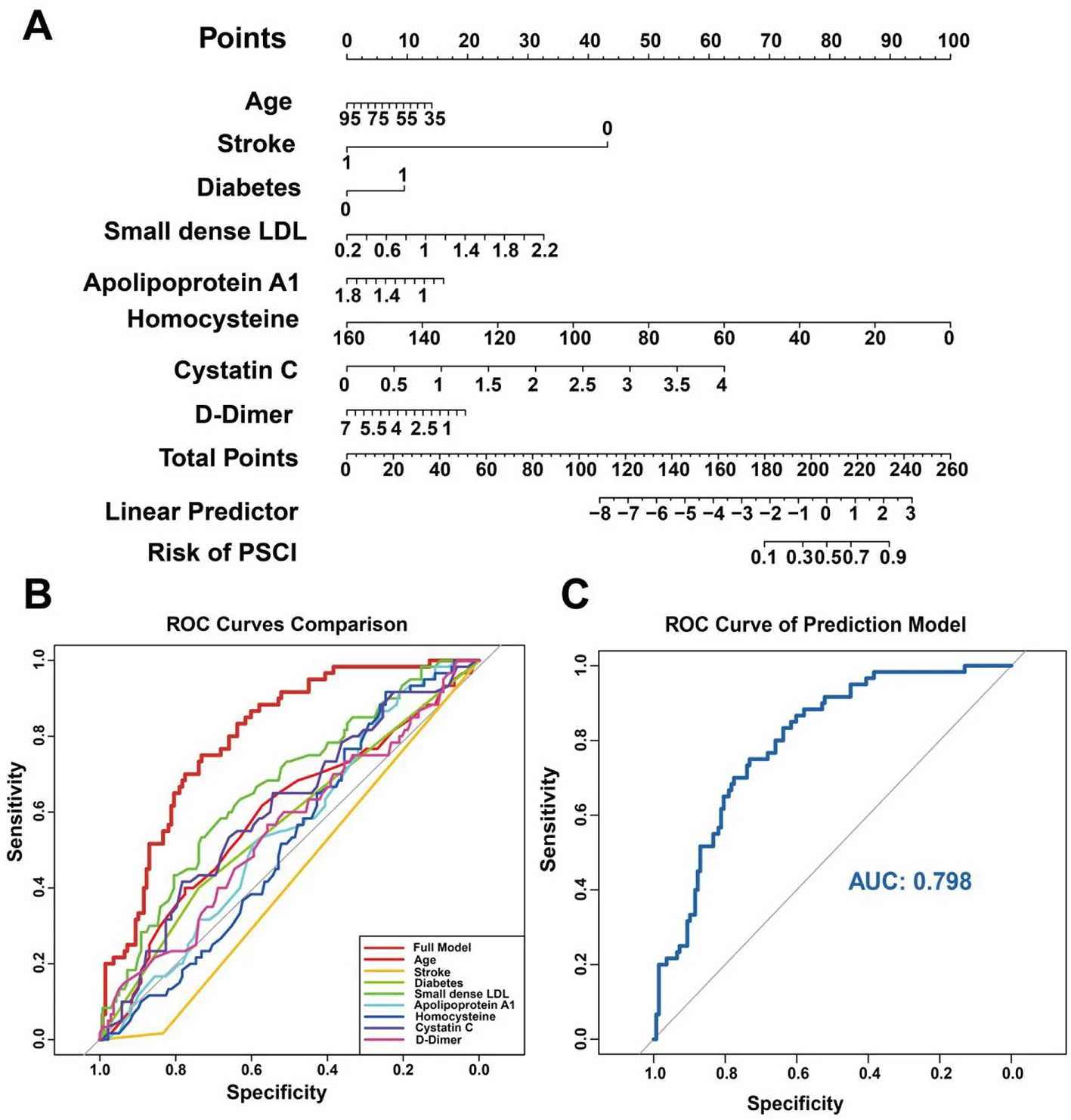
Development and performance evaluation of the prediction model for PSCI. **(A)** Nomogram constructed from the multivariable logistic regression model for individualized prediction of PSCI risk. Each predictor is assigned a corresponding point value, and the sum of individual points yields the total point score, which is converted into the predicted probability of PSCI. Binary variables were coded as 0 (absent) and 1 (present). **(B)** Comparison of receiver operating characteristic (ROC) curves between the full prediction model and individual predictors. The ROC curves demonstrate the discriminative performance of the combined model compared with each single predictor. **(C)** ROC curve of the prediction model showing its overall discrimination ability for PSCI. The area under the curve (AUC) represents the probability that the model correctly distinguishes patients with and without PSCI.

The discriminative performance of the prediction model was evaluated using receiver operating characteristic (ROC) curve analysis. The combined prediction model demonstrated good discrimination for PSCI, with an area under the curve (AUC) of 0.798 (Figure 5C). Compared with individual predictors, the full model showed improved discriminative ability, exceeding the predictive performance of single variables, including stroke history, diabetes mellitus, sdLDL, apolipoprotein A1, homocysteine, cystatin C, and D-dimer (Figure 5B).

### 3.5 Internal validation and clinical utility of the prediction model

Internal validation was performed using 1,000 bootstrap resampling iterations to assess the robustness and optimism of the prediction model. The bootstrap-corrected ROC analysis demonstrated stable discriminative performance, with a mean AUC of 0.777 (95% CI, 0.733–0.802). After adjustment for optimism, the corrected AUC was 0.762, with an estimated optimism of 0.036, indicating that the model retained acceptable discrimination after internal validation (Figure 6A).

**Figure 6:**
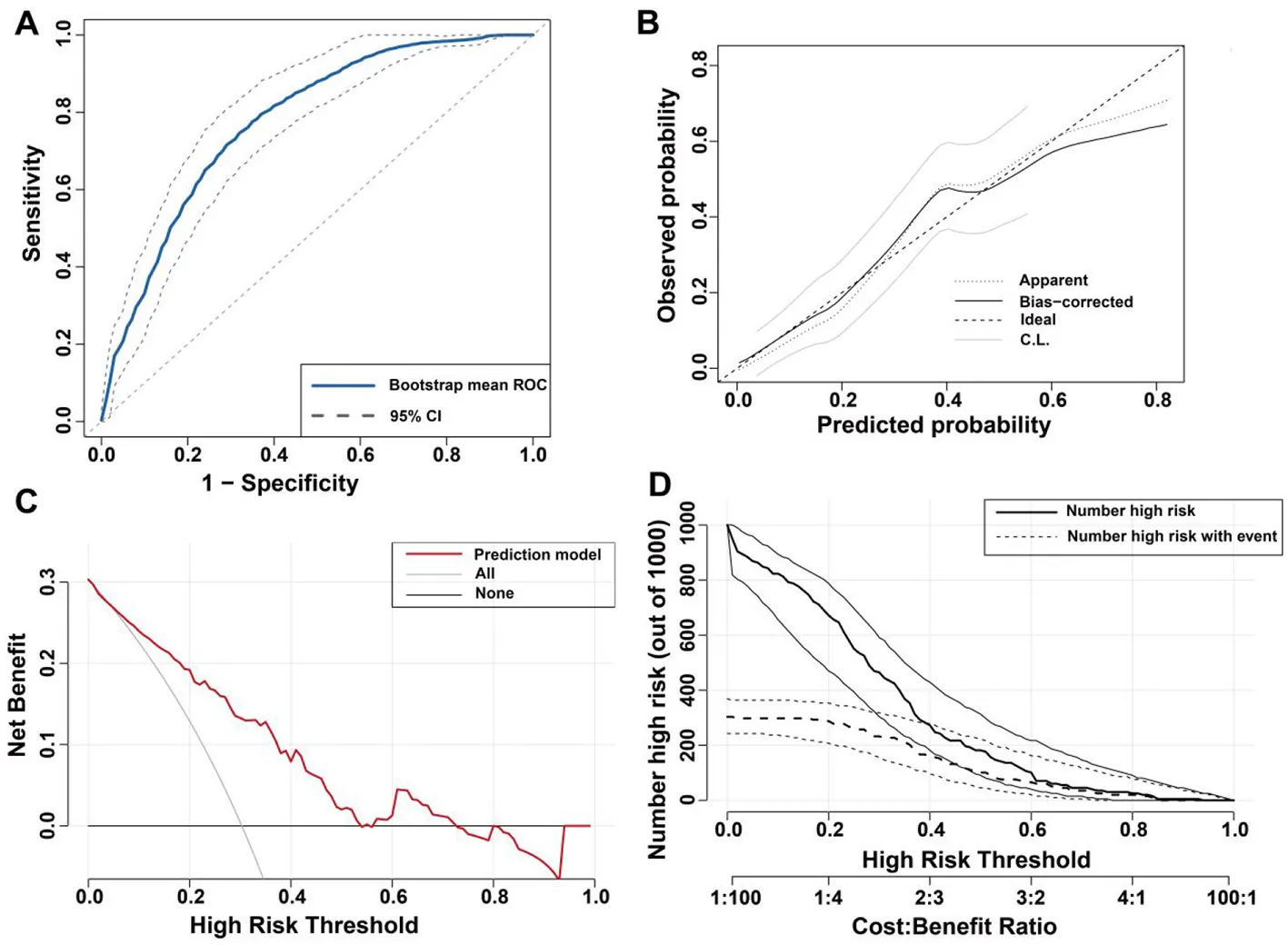
Internal validation and clinical utility assessment of the prediction model for PSCI. **(A)** Bootstrap receiver operating characteristic (ROC) curve of the prediction model based on 1,000 bootstrap resampling iterations. The blue line represents the mean bootstrap ROC curve, and the dashed lines indicate the corresponding 95% confidence interval. **(B)** Calibration curve evaluating the agreement between predicted and observed probabilities. The apparent calibration curve, bias-corrected calibration curve, ideal reference line, and confidence intervals are shown. The proximity of the bias-corrected curve to the ideal line indicates the calibration performance of the model. **(C)** Decision curve analysis (DCA) evaluating the clinical net benefit of the prediction model across different threshold probabilities. The prediction model was compared with the strategies of treating all patients and treating none. **(D)** Clinical impact curve illustrating the relationship between the number of patients classified as high risk and the number of observed PSCI events across different threshold probabilities. The curves demonstrate the potential clinical consequences of applying the prediction model for risk stratification. Abbreviations: ROC, receiver operating characteristic; DCA, decision curve analysis.

Calibration analysis demonstrated good agreement between predicted and observed probabilities. The calibration curve showed that the bias-corrected predictions were closely aligned with the ideal reference line. The calibration intercept was approximately 0 and the calibration slope was 1.00, suggesting that the model predictions were well aligned with the observed outcomes in the internal validation cohort (Figure 6B).

Decision curve analysis was performed to evaluate the potential clinical utility of the prediction model. Across clinically relevant threshold probabilities, the prediction model showed greater net benefit compared with the “treat-all” and “treat-none” strategies, indicating potential value for individualized risk stratification of PSCI (Figure 6C).

The clinical impact curve further illustrated the potential consequences of model application by comparing the number of patients classified as high risk with the number of observed PSCI events across different threshold probabilities. The observed event distribution showed a similar trend with the predicted high-risk population, supporting the potential clinical applicability of the model for identifying patients at increased risk of PSCI (Figure 6D).

## 4 Discussion

This prospective cohort study developed a prediction model for post-stroke cognitive impairment (PSCI) by integrating clinical characteristics with lipid metabolism–related and other routinely available blood biomarkers. The final model incorporated age, prior stroke history, diabetes mellitus, small dense low-density lipoprotein (sdLDL), apolipoprotein A-I (ApoA-I), homocysteine, cystatin C, and D-dimer. The combined model achieved an apparent AUC of 0.798. Following 1,000 bootstrap resampling iterations, the mean AUC was 0.777 (95% CI, 0.733–0.802), and the optimism-corrected AUC was 0.762, indicating that the model retained acceptable discriminative performance after internal validation. Calibration analysis also suggested reasonable agreement between predicted and observed risks. Overall, these findings support the potential value of combining clinical characteristics and circulating biomarkers for PSCI risk stratification, although external validation in independent populations is required before the model can be considered for broader clinical application.

Age was retained in the final prediction model despite the absence of a significant baseline difference between the PSCI and PSCN groups, suggesting that its contribution may depend on its interaction with the broader clinical and biochemical profile. This is consistent with previous evidence linking older age to an increased risk of PSCI.A recent systematic review and meta-analysis identified age as one of the major risk factors for PSCI, while another meta-analysis including 13,322 patients found that patients who developed PSCI were, on average, older than those without cognitive impairment (28, 29). Age may also capture cumulative cerebral vulnerability rather than exerting an isolated effect. Neuroimaging studies have shown that cerebral atrophy, cerebral microbleeds, and increasing white matter hyperintensity burden are associated with poorer post-stroke cognitive outcomes, and moderate-to-severe white matter hyperintensities remain associated with PSCI even after adjustment for age and stroke severity (30, 31). Therefore, in the present cohort, age may function more as a background marker of accumulated cerebrovascular and neurodegenerative vulnerability within a multivariable framework than as a strong standalone discriminator of PSCI. This may partly explain why age was not significantly different between the PSCI and PSCN groups at baseline but nevertheless showed relatively stable selection in the bootstrap LASSO analysis and was retained in the final prediction model.

Interestingly, prior stroke history was inversely associated with 3-month PSCI risk in our cohort, which differs from previous reports linking cerebrovascular burden to cognitive decline after stroke (32, 33). Previous systematic reviews, meta-analyses, and umbrella reviews have consistently identified prior stroke, recurrent stroke, and multiple cerebrovascular lesions as important risk factors for PSCI and post-stroke dementia (34–36). Prospective cohort data have also shown that recurrent stroke contributes to subsequent dementia risk among stroke survivors (37). These associations are biologically plausible, as repeated ischemic insults may lead to cumulative cerebrovascular damage, enhanced neuroinflammation, blood–brain barrier dysfunction, and impaired neuronal recovery (38). Several factors may explain the divergent finding observed in our cohort. First, patients with previous stroke who survived and completed the 3-month cognitive assessment may represent a selected subgroup with relatively favorable functional recovery, better cognitive reserve, or more stable clinical status, whereas patients with recurrent or severe disabling strokes may be more likely to experience functional dependence, mortality, or loss to follow-up (32, 39). Second, individuals with prior stroke may have received earlier and more intensive secondary prevention, including antiplatelet therapy, vascular risk factor management, and lifestyle interventions, which could potentially reduce subsequent vascular injury and cognitive decline (40). Finally, the relatively small number of patients with prior stroke in our cohort and residual confounding from unmeasured factors, such as lesion characteristics, rehabilitation participation, and premorbid cognition, may have influenced the observed association. Therefore, this finding should be interpreted cautiously as an exploratory observation and requires validation in larger multicenter cohorts with longitudinal cognitive assessment.

Diabetes mellitus was retained in the final prediction model despite the absence of a statistically significant baseline difference between the PSCI and PSCN groups. Previous studies nevertheless support an association between diabetes and poorer post-stroke cognition. Lo et al., (36) using individual participant data from 3,146 patients across eight countries, found diabetes to be strongly associated with poorer cognitive outcomes after stroke. Filler et al. (41) further identified diabetes as a potentially modifiable risk factor for PSCI in a meta-analysis of 89 studies involving 160,783 patients (RR = 1.29, 95% CI 1.14–1.45), independent of age and stroke severity. Wang et al. (29) reported a similar association in a meta-analysis of 13,322 patients (RR = 1.10, 95% CI 1.03–1.17). In addition, Swardfager et al. (42) showed that diabetes was associated with more severe cognitive impairment after stroke, particularly when accompanied by depressive symptoms . Collectively, these findings suggest that diabetes may contribute to post-stroke cognitive vulnerability through chronic vascular and metabolic injury and may add prognostic information when combined with other clinical and biochemical predictors.

Small dense low-density lipoprotein (sdLDL) is a kind of low density lipoprotein, which is the core material basis for atherosclerosis in large arteries and arterioles. The findings of the present study are consistent with those of Pokharel et al., (43) who demonstrated that Small Dense Low-Density Lipoprotein Cholesterol (sdLDL-C) levels are significantly negatively correlated with cognitive function, and that reducing atherogenic lipoproteins such as sdLDL-C may improve both cardiovascular health and late-life cognitive outcomes. Beyond its vascular effects, sdLDL may also directly contribute to neural injury. The myelin sheath is a critical structure for maintaining axonal integrity and efficient neural signal transmission. In chronic cerebral ischemia, aberrant expression of low-density lipoprotein receptors (LDLRs) in oligodendrocytes disrupts myelin homeostasis through specific signaling pathways, leading to demyelination, impaired neural conduction, and subsequent cognitive decline (44). In addition, Feng et al. demonstrated that sdLDL and other atherogenic lipoproteins activate astrocyte-mediated inflammatory pathways via LDLR signaling, thereby modulating NLR family pyrin domain containing protein 3 (NLRP3) inflammasome–associated neurotoxicity. This mechanism represents a central link between lipid metabolic dysregulation and post-stroke neuroinflammation (45). Taken together, these findings suggest that sdLDL may be linked to PSCI through both cerebrovascular injury and lipid-related neuroinflammatory pathways, providing biological plausibility for the positive association observed in our cohort.

Apolipoprotein A-I (ApoA-I), the principal structural and functional component of high-density lipoproteins, plays a key role in reverse cholesterol transport and has been implicated in amyloid-independent neurodegenerative processes (46). Genetic variations in the ApoA-I gene may alter protein function, disrupt cholesterol homeostasis in the brain, and thereby increase the risk of cognitive impairment (47). ApoA-I was retained in the final prediction model despite the absence of a significant baseline difference between the PSCI and PSCN groups. Previous studies examining the relationship between ApoA-I and cognitive impairment have yielded inconsistent findings (48). However, conflicting evidence exists. Ma et al. reported that higher serum ApoA-I levels were significantly associated with cognitive impairment in patients with type 2 diabetes mellitus (49), while Deng et al., (50) using machine-learning approaches, found that elevated ApoA-I levels were associated with an increased risk of mild cognitive impairment. In contrast, other studies have reported no significant association between ApoA-I and cognitive impairment (51). These inconsistencies may arise from heterogeneity in ApoA-I functional isoforms, differences in disease types and stages, and variations in study populations and methodologies. The mechanistic relationship between ApoA-I and cognitive impairment therefore remains incompletely understood. Emerging evidence provides insight into potential biological mechanisms underlying this association. After peripheral nerve injury, compared with normal nerves, the concentration of lipoprotein A1 was significantly increased, indicating that lipoprotein A1 may be related to nerve injury (52). Hao et al. (53) and Handattu et al. (54) found that ApoA-I-based nanodisks and ApoA-I-mimetic peptides can reduce amyloid-β deposition, attenuate microgliosis, ameliorate neuronal injury, and improve cognitive performance in Alzheimer's disease models, likely through anti-inflammatory mechanisms. Peripheral ApoA-I synthesized in the liver and intestine can access the central nervous system by crossing the blood–cerebrospinal fluid barrier, and plasma ApoA-I can also be transported into peripheral nerves to promote axonal regeneration and myelin repair (52, 55, 56). Moreover, bidirectional communication between the central nervous system and the liver allows coordinated regulation of hepatic lipid metabolism and lipoprotein synthesis (56). Collectively, these findings suggest several potential mechanisms through which ApoA-I may influence cognitive outcomes, including anti-inflammatory effects, neural repair, and regulation of lipid homeostasis.

Homocysteine (HCY) has been widely investigated as a potential biomarker of post-stroke cognitive impairment (PSCI). A recent meta-analysis of blood biomarkers found that HCY levels were significantly higher in patients with PSCI than in those without PSCI (57). Li et al. (58) reported that HCY had modest discriminative ability for cognitive impairment after progressive ischemic stroke, with an AUC of 0.673. Zhou et al. (59) further showed that the association between elevated HCY and post-stroke cognitive impairment was age-dependent and was stronger in patients younger than 65 years. In addition, Li et al. (60) reported that the relationship between HCY and PSCI varied by sex and follow-up time, with elevated HCY being associated with a higher 12-month PSCI risk in women but not with 3-month PSCI. In contrast, our cohort showed lower HCY levels in the PSCI group, and HCY was inversely associated with PSCI in the multivariable model. This finding should not be interpreted as a protective effect. Rather, it may reflect differences in population characteristics, age and sex distribution, timing of assessment, or unmeasured and residual confounding related to renal function and nutritional status. Mechanistically, elevated HCY has been linked to vascular injury, oxidative stress, infarct burden, and impaired executive function after stroke (61), supporting a biologically plausible adverse effect on cognition. Therefore, the inverse association observed in our study requires confirmation in larger external cohorts.

Although serum CysC levels did not differ significantly between the PSCI and PSCN groups at baseline, CysC was retained in the final model and showed a positive association with PSCI. Previous studies have similarly linked higher CysC levels with cognitive dysfunction and cerebral small-vessel disease. The findings of the present study are consistent with those reported by Zhang et al., (62) demonstrating that serum CysC levels are independently associated with cognitive dysfunction. However, accumulating evidence suggests a more complex role of CysC in post-stroke cognition. Guo et al. (63) proposed that CysC may function as an endogenous neuroprotective factor, with alterations in CysC levels associated with post-stroke cognitive dysfunction. Similarly, Yang et al. (64) reported that exogenous CysC administration reduced infarct volume following ischemic brain injury. Moreover, some studies have described a U-shaped association between CysC concentrations and long-term risk of cognitive impairment, indicating that both excessively high and abnormally low levels may be detrimental (65). In this context, elevated CysC levels may reflect renal dysfunction and vascular injury, thereby increasing the risk of post-stroke cognitive impairment, whereas insufficient CysC levels may weaken endogenous neuroprotection and also contribute to cognitive decline. Therefore, the impact of CysC on post-stroke cognitive function appears to be bidirectional, resembling a “double-edged sword.” CysC thus serves not only as a biomarker of vascular injury but also as an endogenous neuroprotective molecule.

D-dimer, a degradation product of cross-linked fibrin, reflects activation of coagulation and fibrinolysis and may serve as a marker of thrombotic and microvascular burden rather than a direct neurotoxic factor. Stott et al. (66) reported in the PROSPER cohort that higher D-dimer levels were associated with faster cognitive decline in older adults (66), while Gillett et al. (67) found in the REGARDS study that elevated D-dimer was associated with incident cognitive impairment (67). Carcaillon et al. (68) and Quinn et al. (69) further showed that higher D-dimer levels were associated with an increased risk of vascular dementia. Mechanistically, elevated D-dimer may reflect persistent fibrin turnover and a prothrombotic state, which can promote cerebral small-vessel injury, microvascular obstruction, and chronic hypoperfusion. Tomimoto et al. (70) also linked coagulation–fibrinolysis activation with severe leukoaraiosis and subcortical vascular dementia, supporting a possible vascular pathway between hemostatic dysregulation and cognitive decline. In the present study, although baseline D-dimer levels did not differ significantly between the PSCI and PSCN groups, D-dimer was retained in the final multivariable model. This finding suggests that its association with PSCI may be modest when considered alone but may become more informative in the presence of other vascular and metabolic abnormalities. D-dimer may therefore reflect an underlying prothrombotic and microvascular environment that contributes to post-stroke cognitive vulnerability, although this interpretation requires confirmation in larger external cohorts.

The prediction model developed in this study showed acceptable discriminative performance, with an apparent AUC of 0.798. After 1,000 bootstrap resampling iterations, the mean AUC was 0.777 (95% CI, 0.733–0.802), and the optimism-corrected AUC was 0.762, suggesting that the model retained reasonable discrimination after internal validation. The nomogram provides an intuitive representation of the multivariable model and may facilitate individualized estimation of PSCI risk. Importantly, the model incorporates routinely available clinical characteristics and blood-based biomarkers, including age, stroke history, diabetes mellitus, sdLDL, ApoA-I, homocysteine, cystatin C, and D-dimer, which may support its practical use in clinical risk assessment without the need for specialized imaging or testing. Calibration analysis suggested reasonable agreement between predicted and observed risks, while decision-curve and clinical-impact analyses provided preliminary evidence of potential clinical utility. However, given the single-center design and the absence of external validation, the model should currently be regarded as a preliminary clinical prediction tool rather than one ready for widespread implementation. Further validation in larger, independent, and more diverse populations is required before its broader clinical application.

Several limitations should be acknowledged. First, the single-center design limits the generalizability of our findings to external populations, different geographic regions, and diverse ethnic groups. The relatively high proportion of PSCI cases in our cohort (138/198, 69.7%) may have influenced model performance estimates, particularly the positive predictive value, which is dependent on outcome prevalence. Therefore, the predictive value of the nomogram may differ in populations with lower PSCI incidence and requires further validation in cohorts with diverse baseline risks. Second, cognitive function was assessed only at 3 months post-stroke, and the absence of longitudinal follow-up limits evaluation of long-term predictive performance and biomarker effects on PSCI progression. Additionally, only patients who survived and completed the 3-month assessment were included, potentially introducing selection and survivor bias. Moreover, the cohort mainly included patients with mild stroke (admission NIHSS < 3), which may limit the generalizability of the model to patients with more severe stroke. Further validation in broader stroke populations is needed. Several non-biological factors, including education, psychological status, socioeconomic conditions, sleep quality, rehabilitation participation, and cognitive reserve, were not evaluated and should be considered in future models (71, 72). Unmeasured premorbid, neuropsychiatric, and imaging factors may also have influenced cognition; therefore, T-MoCA should be interpreted as a screening measure rather than a definitive diagnosis (72). Moreover, the use of T-MoCA may limit assessment of visuospatial and executive domains and lead to misclassification of subtle cognitive deficits, highlighting the need for validation against comprehensive neuropsychological batteries. Although sex was recorded, handedness was not collected, preventing assessment of its potential influence on cognitive performance (73). Future multicenter studies with larger samples and longitudinal follow-up are warranted to improve the generalizability, predictive accuracy, and clinical translation of PSCI models.

## 5 Conclusion

In conclusion, this study developed a multivariable prediction model for post-stroke cognitive impairment incorporating age, prior stroke history, diabetes mellitus, small dense low-density lipoprotein, apolipoprotein A1, homocysteine, cystatin C, and D-dimer. The model combined routinely available clinical characteristics with circulating biomarkers and showed acceptable discriminative performance, with an apparent AUC of 0.798 and an optimism-corrected AUC of 0.762 after bootstrap internal validation. The nomogram may provide a practical approach for individualized estimation of PSCI risk after acute ischemic stroke. These findings support the potential utility of the model for PSCI risk stratification, although external validation is required before clinical implementation.

## Data Availability

The raw data supporting the conclusions of this article will be made available by the authors, without undue reservation.
