# Supplementary material for: A clinical and biomarker model for predicting post-stroke cognitive impairment: a prospective cohort study
Source: Front Neurol. 2026 Oct 14;17:1963734. doi: 10.3389/fneur.2026.1963734 (PMC13713335; doi:10.3389/fneur.2026.1963734)
Supplement: Supplementary file 1 [file Table_1.docx]

Supplementary Table 1

| Variable_level | OR | OR (95% CI) | P value |
| --- | --- | --- | --- |
| Sex | 0.753 | 0.753 (0.401–1.426) | 0.3787 |
| Thrombolysis | 1.318 | 1.318 (0.590–2.838) | 0.4874 |
| Thrombectomy | 0.56 | 0.560 (0.083–2.321) | 0.4725 |
| Stroke history | 0.085 | 0.085 (0.005–0.418) | 0.017 |
| Hypertension | 1.494 | 1.494 (0.799–2.853) | 0.2148 |
| Diabetes mellitus | 1.889 | 1.889 (0.991–3.588) | 0.0519 |
| Coronary heart disease | 0.687 | 0.687 (0.187–2.040) | 0.527 |
| Dyslipidemia | 0.373 | 0.373 (0.020–2.249) | 0.3661 |
| Atrial fibrillation | 0.917 | 0.917 (0.129–4.392) | 0.9192 |
| Smoking history | 1.64 | 1.640 (0.672–3.861) | 0.2624 |
| Alcohol consumption | 1.525 | 1.525 (0.536–4.088) | 0.4084 |
| Surgical history | 0.752 | 0.752 (0.326–1.617) | 0.4805 |
| Antiplatelet agents | 0.798 | 0.798 (0.332–1.777) | 0.5937 |
| Anticoagulants | 0.275 | 0.275 (0.015–1.550) | 0.2291 |
| Antihypertensive drugs | 1.187 | 1.187 (0.647–2.192) | 0.5802 |
| Hypoglycemic drugs | 1.311 | 1.311 (0.659–2.556) | 0.4314 |
| TOAST classification（Small-vessel occlusion） | 1.142 | 1.142 (0.522–2.416) | 0.7319 |
| TOAST classification（Cardioembolism） | 0.124 | 0.124 (0.007–0.633) | 0.0457 |
| OCSP classification（Partial anterior circulation） | 1.298 | 1.298 (0.600–2.904) | 0.514 |
| OCSP classification（Posterior circulation） | 1.46 | 1.460 (0.641–3.407) | 0.3722 |
| OCSP classification（Lacunar） | 0.569 | 0.569 (0.028–3.983) | 0.6217 |
| Age | 0.973 | 0.973 (0.941–1.005) | 0.1002 |
| Admission NIHSS score | 0.977 | 0.977 (0.890–1.062) | 0.6052 |
| Admission GCS score | 1.341 | 1.341 (0.915–3.151) | 0.329 |
| Systolic blood pressure | 0.992 | 0.992 (0.976–1.008) | 0.3267 |
| Diastolic blood pressure | 0.98 | 0.980 (0.951–1.008) | 0.1653 |
| Body mass index (BMI) | 0.968 | 0.968 (0.890–1.050) | 0.4332 |
| Small dense LDL (sdLDL) | 4.629 | 4.629 (2.046–10.976) | 0.0003 |
| Apolipoprotein B (ApoB) | 8.674 | 8.674 (2.173–37.876) | 0.0029 |
| Apolipoprotein A1 (ApoA1) | 0.295 | 0.295 (0.051–1.586) | 0.1621 |
| Apolipoprotein E (ApoE) | 1.059 | 1.059 (0.874–1.273) | 0.5461 |
| Triglycerides | 1.434 | 1.434 (1.065–1.968) | 0.0197 |
| Low-density lipoprotein cholesterol (LDL-C) | 1.749 | 1.749 (1.231–2.533) | 0.0023 |
| High-density lipoprotein cholesterol (HDL-C) | 0.677 | 0.677 (0.195–2.221) | 0.5273 |
| Total cholesterol | 1.344 | 1.344 (1.018–1.792) | 0.0391 |
| Homocysteine | 0.97 | 0.970 (0.928–0.999) | 0.105 |
| Folate | 1.011 | 1.011 (0.987–1.036) | 0.37 |
| Vitamin B12 | 0.999 | 0.999 (0.996–1.000) | 0.1229 |
| Glycated hemoglobin (HbA1c) | 1.112 | 1.112 (0.924–1.333) | 0.2508 |
| Fasting glucose | 1.073 | 1.073 (0.957–1.201) | 0.2182 |
| D-Dimer | 0.761 | 0.761 (0.478–1.064) | 0.1705 |
| Fibrinogen | 1.423 | 1.423 (0.979–2.106) | 0.0698 |
| Cystatin C (CysC) | 1.934 | 1.934 (0.794–5.125) | 0.1552 |
| C-reactive protein (CRP) | 0.974 | 0.974 (0.920–1.001) | 0.2741 |

Supplementary Table 2

| Predictor | Unit_or_reference | Beta | SE | OR | CI_low | CI_high | OR (95% CI) | P value |
| --- | --- | --- | --- | --- | --- | --- | --- | --- |
| Age | per 1 year | -0.019 | 0.019 | 0.981 | 0.946 | 1.018 | 0.981 (0.946–1.018) | 0.307 |
| Prior stroke | Yes vs No | -3.529 | 1.396 | 0.029 | 0.002 | 0.452 | 0.029 (0.002–0.452) | 0.01147 |
| Diabetes mellitus | Yes vs No | 0.776 | 0.382 | 2.174 | 1.027 | 4.6 | 2.174 (1.027–4.600) | 0.04235 |
| sdLDL | per 1 mmol/L | 1.334 | 0.482 | 3.794 | 1.475 | 9.762 | 3.794 (1.475–9.762) | 0.005677 |
| ApoA1 | per 1 g/L | -1.31 | 1.081 | 0.27 | 0.032 | 2.247 | 0.270 (0.032–2.247) | 0.2258 |
| Homocysteine | per 1 μmol/L | -0.051 | 0.024 | 0.95 | 0.907 | 0.995 | 0.950 (0.907–0.995) | 0.03061 |
| Cystatin C | per 1 mg/L | 1.278 | 0.594 | 3.59 | 1.121 | 11.499 | 3.590 (1.121–11.499) | 0.03142 |
| D-dimer | per 1 mg/L | -0.229 | 0.218 | 0.795 | 0.519 | 1.218 | 0.795 (0.519–1.218) | 0.2926 |
